# COVID-19 and Laparoscopic Surgery: Scoping Review of Current Literature and Local Expertise

**DOI:** 10.2196/18928

**Published:** 2020-06-23

**Authors:** Robert Adrianus de Leeuw, Nicole Birgit Burger, Marcello Ceccaroni, Jian Zhang, Jurriaan Tuynman, Mohamed Mabrouk, Pere Barri Soldevila, Hendrik Jaap Bonjer, Pim Ankum, Judith Huirne

**Affiliations:** 1 Amsterdam University Medical Center Vrije Universiteit Amsterdam Amsterdam Netherlands; 2 Department of Obstetrics and Gynecology, Gynecologic Oncology and Minimally-Invasive Pelvic Surgery International School of Surgical Anatomy Istituto Di Ricovero e Cura a Carabettere Scientifico Sacro Cuore Don Calabria Hospital, Negrar di Valpolicella Verona Italy; 3 Department of Obstetrics and Gynecology International Peace Maternity and Child Health Hospital School of Medicine, Shanghai Jiaotong University Shanghai China; 4 Department of Surgery Amsterdam University Medical Center Vrije Universiteit Amsterdam Amsterdam Netherlands; 5 Cambridge Endometriosis and Endoscopic Surgery Unit Cambridge University Hospitals NHS Foundation Trust Addenbrook United Kingdom; 6 Dexeus Mujer Hospital Universitari Dexeus Barcelona Spain; 7 Department of Gynecology and Obstetrics, Research Institute Reproduction and Development Amsterdam University Medical Center Vrije Universiteit Amsterdam Amsterdam Netherlands

**Keywords:** laparoscopy, COVID-19, surgical procedures, operative, corona 2019, surgery, pandemic, outbreak, infectious disease, health care provider, physician

## Abstract

**Background:**

The current coronavirus disease (COVID-19) pandemic is holding the world in its grip. Epidemiologists have shown that the mortality risks are higher when the health care system is subjected to pressure from COVID-19. It is therefore of great importance to maintain the health of health care providers and prevent contamination. An important group who will be required to treat patients with COVID-19 are health care providers during semiacute surgery. There are concerns that laparoscopic surgery increases the risk of contamination more than open surgery; therefore, balancing the safety of health care providers with the benefit of laparoscopic surgery for the patient is vital.

**Objective:**

We aimed to provide an overview of potential contamination routes and possible risks for health care providers; we also aimed to propose research questions based on current literature and expert opinions about performing laparoscopic surgery on patients with COVID-19.

**Methods:**

We performed a scoping review, adding five additional questions concerning possible contaminating routes. A systematic search was performed on the PubMed, CINAHL, and Embase databases, adding results from gray literature as well. The search not only included COVID-19 but was extended to virus contamination in general. We excluded society and professional association statements about COVID-19 if they did not add new insights to the available literature.

**Results:**

The initial search provided 2007 records, after which 267 full-text papers were considered. Finally, we used 84 papers, of which 14 discussed severe acute respiratory syndrome coronavirus 2 (SARS-CoV-2). Eight papers discussed the added value of performing intubation in a low-pressure operating room, mainly based on the SARS outbreak experience in 2003. Thirteen papers elaborated on the risks of intubation for health care providers and SARS-CoV-2, and 19 papers discussed this situation with other viruses. They conclude that there is significant evidence that intubation and extubation is a high-risk aerosol-producing procedure. No papers were found on the risk of SARS-CoV-2 and surgical smoke, although 25 papers did provide conflicting evidence on the infection risk of human papillomavirus, hepatitis B, polio, and rabies. No papers were found discussing tissue extraction or the deflation risk of the pneumoperitoneum after laparoscopic surgery.

**Conclusions:**

There seems to be consensus in the literature that intubation and extubation are high-risk procedures for health care providers and that maximum protective equipment is needed. On the other hand, minimal evidence is available of the actual risk of contamination of health care providers during laparoscopy itself, nor of operating room pressure, surgical smoke, tissue extraction, or CO_2_ deflation. However, new studies are being published daily from current experiences, and society statements are continuously updated. There seems to be no reason to abandon laparoscopic surgery in favor of open surgery. However, the risks should not be underestimated, surgery should be performed on patients with COVID-19 only when necessary, and health care providers should use logic and common sense to protect themselves and others by performing surgery in a safe and protected environment.

## Introduction

### Background

Coronavirus disease (COVID-19) is spreading worldwide, and all health care workers are affected by it [1]. At the moment of writing, the World Health Organization estimated over 2.5 million confirmed cases of COVID-19 and over 175 thousand deaths [2]. It is estimated from the Chinese outbreak that the risk of death is as high as 12% in epicenters of the epidemic and as low as 1% in less severely affected areas. This large difference may be due to a breakdown of the health care system in the epicenter, enhanced public health interventions, and enhanced hygienic measures [3].

According to Médecins Sans Frontières, nearly 1700 healthcare providers have been infected, representing 8% of the total COVID-19 cases in Italy, despite all preventive measures [4]. Therefore, health care providers are the highest risk group for infection, severe illness, and intensive care admission. This stresses the incredible importance of protecting this group.

Due to the combination of increased risk of individual infection and the effects of a breakdown of the healthcare system, it is even more relevant to discuss how to properly protect health care providers. If no personal protective equipment is available, health care workers will be jeopardized [5,6]. Moreover, the shortage of supplies is forcing management to make difficult decisions as to where supplies should be allocated and who needs them most in a hospital.

So, who is at risk? According to the US Centers for Disease Control and Prevention, all health care providers that are in direct contact with infectious secretions from a patient with COVID-19 are at risk. Secretions at risk for viral transmission include sputum, serum, blood, feces, and especially respiratory droplets [7,8]. Health care providers are all recommended to wear personal protective equipment (PPE). The risk increases with exposure to aerosol-generating procedures for at least 10 minutes at a distance of fewer than 2 meters from the patient [9]. Studies have shown that procedures such as endotracheal intubation, extubation, noninvasive ventilation, and exposure to aerosols in an open circuit are associated with high risk of viral transmission. Guidelines about the PPE needed in these situations are receiving increasing attention [10].

According to Wong et al [11], the main risk groups in the operating theater are those who cannot cancel or delay elective procedures. Foremost, of course, are anesthesiologists; however, departments such as intervention radiology, obstetrics, and cardiothoracic surgery are also at risk. Many acute surgical interventions are performed by laparoscopy; however, very little is written about the risks for health care providers of performing laparoscopic surgery on a patient with COVID-19. There is a debate in the literature whether open surgery is safer for health care providers compared to laparoscopic surgery [12,13].

The objective of this study is to provide an overview of potential contamination routes and possible risks for health care providers, and propose research questions based on current literature and expert opinions about laparoscopic surgery on patients with COVID-19.

### Theoretical Contamination Routes During Laparoscopic Surgery

Before we can elaborate on the theoretical contamination routes, we must first discuss the contamination agents. The agents of contamination can be divided into three groups: those with proven infectious transmission, such as droplets, close contact, and aerosol transmission [14]; those with proven RNA presence, but no proven contamination yet, such as feces, inanimate surfaces, and blood [8,15,16]; and unknown or highly debated agents or even the presence of RNA, such as urine and amniotic fluid [8]. It should be noted that many studies are underway to determine which of these agents are, in addition to containing virus RNA, are also infectious. Taking these agents into consideration, there are several theoretical contamination routes by which health care providers can be infected by a COVID-19 positive patient.

**Figure 1:**
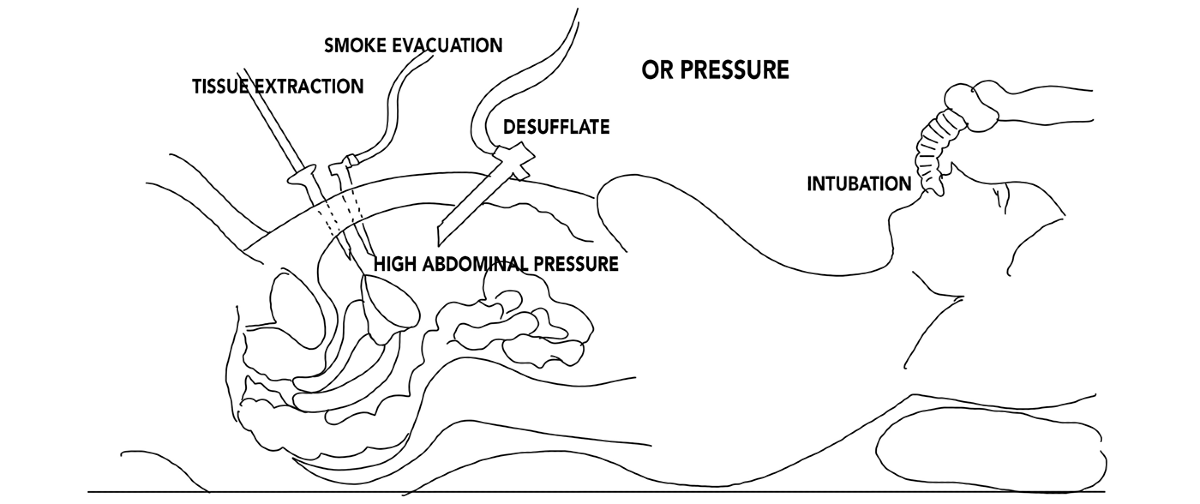
Contamination routes during laparoscopy. OR: operating room.

Figure 1 shows potential viral contamination routes in the IR during laparoscopic surgery. The first and most discussed contamination route is intubation and extubation [17]. At this moment, the patient will excrete the most virulent respiratory secretions. The second risk is smoke and air evacuation during surgery [18]. During laparoscopy, smoke and aerosols are generated, not only by cauterization of blood vessels but also by dissection. This smoke can contain virulent DNA and RNA and is sometimes evacuated directly into the overpressured operating room (OR) by opening a valve on a trocar. The third contamination risk is tissue extraction [19]. Removing tissue, such as an appendix, bowel segment, gallbladder, cyst, or ectopic pregnancy, can cause excretions to be expelled from the body; the higher abdominal pressure from laparoscopy creates aerosols from excretions such as blood and mucus. The fourth moment at risk for contamination is at the end of the surgery, when the abdominal pressure is released by desufflation [19]. All the air, possibly filled with virulent DNA and RNA, is released into the air of the OR, usually under relatively high pressure. A fifth risk factor can be the positive air pressure in the OR, which pushes aerosols out of the OR into hallways and other ORs [17].

## Methods

To provide insight into the possible risks of the abovementioned contaminating routes, we believe a scoping review is most suited. A scoping review allows a broader search and answers multiple questions while still performing a systematic search [20]. Because we expected few results from a search on COVID-19 and laparoscopy, we performed five additional searches for the contamination route and viruses in general.

### Systematic Search

The literature search was performed on April 24, 2020, by searching the PubMed, CINAHL, and Embase databases. We then added gray literature from Google Scholar and local expertise and handbooks from the authors themselves from China, Italy, Spain, the United Kingdom, and the Netherlands. The search string can be found in Multimedia Appendix 1. The five additional questions were:

1. What is the effect of operating room pressure on the contamination risk of COVID-19?
2. What is known about the additional risk during intubation and extubation?
3. Does smoke evacuation during laparoscopic surgery increase the risk of the spread of COVID-19 particles?
4. Is anything known about tissue extraction during laparoscopic surgery on a patient with COVID-19?
5. Does desufflation of the abdomen after laparoscopic surgery create airborne aerosols that endanger health care providers?

### Inclusion Criteria

Types of studies included were trials, reviews, case studies or series, and other descriptive studies concerning contamination of health care providers during (laparoscopic) surgery in the operating theater. We also included expert opinions if they added additional insight to the current literature.

### Exclusion Criteria

We excluded society and professional association statements about COVID-19 if they did not add any new information. We did use them to snowball their references. We also excluded commentaries such as letters to the editor and papers not written in English.

### Study Selection

Working independently and in duplicate, reviewers RDL and NB screened all record titles and abstracts. Potentially eligible abstracts and abstracts with disagreement or insufficient information were screened in full text. Disagreements were addressed by discussion of the full text.

## Results

### Literature Search

**Figure 2:**
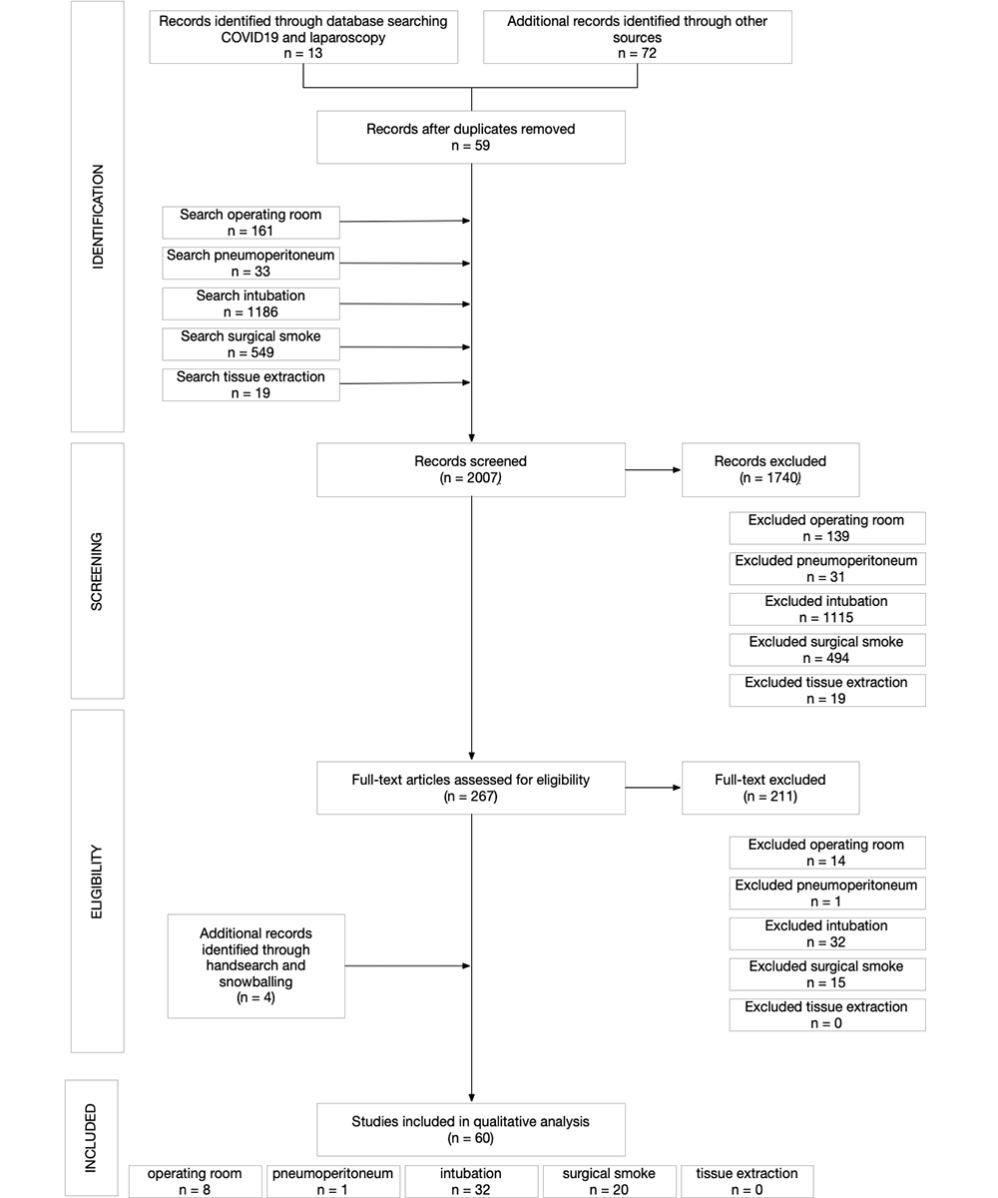
Preferred Reporting Items for Systematic Reviews and Meta-Analyses flow diagram of the literature search and results.

Figure 2 shows a flowchart of the literature search and results. The initial search identified 2007 records, of which 59 concerned COVID-19. After excluding 1740 records based on their title and abstract, we assessed 267 full-text papers for eligibility. Papers were excluded because they discussed a treatment therapy or diagnostic method (118/267, 44.2%), did not provide any new information (society statements, letters to the editor and others) (30/267, 11.2%), were not related to our question (12/267, 4.5%) or were not available in English (9/267, 3.4%). After hand-searching the papers and society statements, we were left with 60 papers for this review. Of these 60 papers, 21 (35%) concerned COVID-19, and 39 (65%) discussed our questions in regard to other viral transmissions. We will now discuss the results for each of the five proposed questions.

### 1. What is the Effect of Operating Room Pressure on the Contamination Risk of COVID-19?

We found 8 papers discussing the effects of OR safety and the spread of virus DNA. Only 1 paper actually discussed the experience with COVID-19 in Wuhan [11], and all studies were based on theoretical risks (see Table 1).

**Table 1:** Literature reports concerning viral transmission in operating rooms.

Study	Country of study	Design	Location and year of evaluation	Pathogen evaluated	Study quality (GRADE^a^)
Zhao et al [21]	China	Retrospective cohort study	Wuhan 2020	SARS-CoV-2^b^	Low
Pei et al [22]	China	Case-control study	Peking 2003	SARS^c^	Low
Kamming et al [23]	Canada	Experience paper	Toronto 2003	SARS	Low
Chee et al [24]	Singapore	Experience paper	Singapore 2003	SARS	Low
Tien et al [25]	Canada	Case series	Toronto 2003	SARS	Low
Park et al [26]	South Korea	Experience paper	Sungkyunjkwan 2015	MERS^d^	Low
Beasley et al [27]	United States	Opinion paper	Washington 2004	Smallpox	Low
Santos de Silva et al [28]	Brazil	Case report	Vale dos Sinos 2014	Adenovirus	Low

^a^GRADE: Grading of Recommendations, Assessment, Development, and Evaluations.

^b^SARS-CoV-2: severe acute respiratory syndrome coronavirus 2.

^c^SARS: severe acute respiratory syndrome.

^d^MERS: Middle Eastern respiratory syndrome.

An OR with a negative pressure environment is ideal to reduce dissemination of the virus by preventing air from escaping the OR [11]. Both the Society of American Gastrointestinal and Endoscopic Surgeons (SAGAS) and the American Society of Gastrointestinal Endoscopy advise that surgery be performed in negative pressure ORs [29,30]. However, a standard OR is usually designed to be at positive pressure relative to the surrounding air. Tien et al [25] reported that during the severe acute respiratory syndrome (SARS) outbreak, surgical procedures were performed within airborne isolation Intensive Care Unit rooms and with additional PPE precautions. This eliminated the risk of intrafacility transport and avoided the need to make environmental modifications to the operating room. Other papers discuss the same contamination route with SARS and Middle Eastern respiratory syndrome (MERS) [22-24,26]. Beasley et al [27] discussed even more isolation strategies in the case of surgery on patients with smallpox.

In Singapore, dedicated separate ORs for surgery on patients with COVID-19 have been installed. The aim was to reduce the risk of contamination of other ORs and patients. Each OR had its own ventilation system with an integrated high-efficiency particulate air (HEPA) filter. The traffic and flow of contaminated air were minimized by locking all doors to the OR during surgery, with only one possible route for entry and exit via the scrub room [11].

Wax et al [31] provided practical recommendations to decrease viral spread when managing a patient infected with COVID-19. Their advice is to convert operating rooms to negative pressure environments with airflow changes.

### 2. What is Known About the Additional Risk During Intubation and Extubation?

Thirteen papers were found discussing intubation and extubation of patients with COVID-19 (see Table 2). Another 19 papers discuss the risk of intubation for health care providers for viruses other than severe acute respiratory syndrome coronavirus 2 (SARS-CoV-2, Multimedia Appendix 2).

**Table 2:** Literature concerning intubation and SARS-CoV-2 virus in 2020.

Study	Region	Design	Main topic or result
Cook [32]	United Kingdom	Narrative review	Purpose and use of PPE^a^
Wax [31]	Canada	Review	Anesthesia guidelines
Heinzerling [33]	United States	Case series	3/121 (24.8%) of health care professionals tested positive
Meng [34]	China	Experience paper	29% of hospitalized COVID-19^b^ patients were health care providers
Sorbello [35]	Italy	Experience paper	High level PPE for aerosol-generating procedures
Yao [36]	China	Experience paper	Anesthesia advice for intubation
Zhao [21]	China	Retrospective cohort study	Anesthetic management guidelines
Zuo [37]	China	Experience paper	Anesthesia guidelines
Giwa [38]	Italy	Experience paper	Complete COVID-19 overview
Greenland [39]	United States	Review	Intubation advice
Kim [40]	South Korea	Expert opinion	Anesthesia advice
Au Yong [41]	Singapore	Experience paper	Intubation advice
Zhang [42]	China	Case series	No health care providers infected

^a^PPE: personal protective equipment.

^b^COVID-19: coronavirus disease.

Two reviews from Cook et al [32] and Wax et al [31] provide a great overview of current knowledge and stress the increased risk to health care providers during intubation and extubation. A case series by van Heinzerling [33] shows that 3/121 health care providers (2.5%) tested positive after assisting intubation.

Zucco at al [43] warn that the anesthesia professionals and intensivists have the highest risk of exposure to respiratory droplets during intubation and extubation. They provide a 10-point list of precautions that should be taken into account when intubating or extubating patients with COVID-19 [44]. Again, Wax et al [31] advise that high-risk aerosol-generating procedures, such as intubation, not be performed in a positive pressure environment. Won et al [11] advise the use of at least a National Institute for Occupational Safety and Health (NIOSH)-certified N95 respirator, eye protection (either goggles or a full face shield), cap, gown, and gloves. As transmission remains possible despite N95 protection, staff participating in aerosol-generating procedures can wear a powered air purifying respirator (PAPR). Repici et al [45] suggest additional PPE during endoscopic procedures but does not provide additional insight into the risks of intubation.

Learning from other experiences, 16 studies stress the increased risk for health care providers during intubation from the 2003 SARS period (Multimedia Appendix 2). Pei et al [22] show that the odds ratio (OR) that a health care provider will be infected is 30.8. While others show lower numbers (Rabout et al [46] 2.79 and Tran et al [47] 6.6), they all label intubation as a very high-risk procedure for health care providers.

### 3. Does Smoke Evacuation During Laparoscopic Surgery Increase the Risk of the Spread of COVID-19 Particles?

We found 25 papers discussing the effects of surgical smoke on health care providers. However, none of these papers is specific to COVID-19. A review from Mowbay et al [48] from 2013 included 20 studies and showed the diverse outcomes of these studies; they concluded that infective virus DNA can be found in the smoke plume, but the risk to OR staff is unproven. We found 19 studies not mentioned in the Mowbay review (see Table 3) that also showed diverse results. In Korea, Kwak et al [49] found hepatitis B DNA in surgical smoke in 10/11 cases; however, Waynandt [50] did not find any human papillomavirus (HPV) in 28 cases of CO_2_ laser plume. However, another study [51] shows that laparoscopic surgery is associated with better preservation of the immune system than open surgery. This results in a decreased incidence of infectious complications. A systematic review concerning surgical smoke during open surgery [48] shows that in terms of infection risk, 6/20 (30%) of the studies assessed surgical smoke for the presence of viruses, with only 1 study (5%) positively identifying viral DNA in laser-derived smoke. This has been shown for HPV DNA [52,53].

**Table 3:** Literature concerning surgical smoke plumes.

Study	Country, year	Design	Pathogen evaluated	Type of smoke	Positive results
Mowbray et al [48]	Multiple, 2013	Systematic review	HPV^a^, compounds, cells, particles	Diathermy, laser, ultrasonic-derived smoke	20 studies included
Subbarayan et al [54]	United States, 2019	Case series	HPV16	Laparoscopic electrosurgery	0/6 cases
Neumann et al [55]	Germany, 2017	Prospective pilot series	HPV	Loop electrosurgical excision procedure	4/24 cases
Dodhia et al [56]	United States, 2017	Case series	HPV	KTP laser	0/12 fibers
Kashima et al [57]	United States, 2016	Case series	HPV	CO_2_ laser	17/30 cases
Garden et al [58]	United States, 2015	Animal study	Papillomavirus	CO_2_ laser	3/3 cases
Kwak et al [49]	Korea, 2014	Case series	Hepatitis B	Laparoscopic electrosurgery	10/11 cases
Manson [59]	United States, 2013	Review	HPV	CO_2_ laser	4 studies included
Weynandt et al [50]	Germany 2010	Case series	HPV	CO_2_ laser, argon plasma	0/28 cases
Taravella et al [60]	United States, 1998	Experiment	Polio virus	Excimer laser	2/2 cases
Hughes et al [61]	United States, 1997	Case series	HPV	Erbium YAG laser	0/5 cases
Hagen et al [62]	United States, 1997	Experiment	Pseudorabies virus	Excimer laser	0/20 cases
Gloster et al [63]	United States, 1995	Survey	HPV	CO_2_ laser	31/570 reports
Jewett et al [64]	United States, 1992	Experiment	Hemoglobin	Drill aerosols	5 of 5 cases
Starr et al [65]	United States, 1992	Experiment	Simian immunodeficiency virus	CO_2_ laser	0 of 5 cases
Baggish et al [52]	United States, 1991	Case series	HIV	CO_2_ laser	0 of 12 cases
Hallmo et al [66]	Norway, 1990	Case report	HPV	Erbium YAG laser	1 of 1 cases
Andre et al [67]	France, 1990	Case report	HPV	CO_2_ laser	2 of 2 cases
Sawchuk et al [68]	United States, 1988	Case series	HPV	CO_2_ laser	4 of 8 cases
Bellina et al [69]	United States, 1982	Experiment	HPV	CO_2_ laser	No viable virus

^a^HPV: human papillomavirus.

### 4. Is Anything Known About Tissue Extraction During Laparoscopic Surgery on a Patient With COVID-19?

We found no studies found concerning this subject. The only studies that we found concerned malignant cells; however, those were out of the scope of this review. One study [70] showed that during laparoscopic surgery, 48.5% of surgeons’ masks, 29.5% of assisting surgeons’ masks, and 31.8% of scrub nurses’ masks were positive for either visible or visually enhanced blood contamination. This demonstrates that wearing masks is of great importance, even when performing laparoscopic surgery.

### 5. Does Desufflation of the Abdomen After Laparoscopic Surgery Create Airborne Aerosols That Endanger Health Care Providers?

One case study discussed the desufflation of CO_2_ gas used during laparoscopic rectal surgery [71]. SAGES recently stated that there is a good possibility of viral contamination during laparoscopy; they added, “While it is unknown whether coronavirus shares these properties, it has been established that other viruses can be released during laparoscopy with carbon dioxide.” However, this has only been shown in smoke, not clear CO_2_ [72].

In one study, the effects of COVID-19 on the strategy for colorectal cancer patients is discussed. The authors especially recommend that natural orifice specimen extraction surgery and transanal total mesorectal excision should be performed with caution during the epidemic period because fecal-oral transmission and aerosol transmission during this type of surgery have not been excluded. A protective stoma should reasonably be carried out, and the protection of OR personnel should be strengthened [73].

## Discussion

There is some existential consensus in the literature that intubation and extubation are high-risk procedures for health care providers. Studies have shown ORs as high as 30, stressing the importance of proper PPE during those procedures [22]. Literature suggests that intubation and extubation should preferably be performed in a low-pressure environment with protective gear for the health care providers. A reasonable number of studies show that surgical smoke contains viral DNA and that health care providers should avoid inhaling it. The infectiousness of tissue extraction and the insufflation gas itself is absolutely unknown, and all advice is at least “arguable” (see Table 4).

When current knowledge does not help us any further, we are faced with a dilemma. Should we follow the conservative route and provide extensive PPE and prevent surgery at all costs? This may sound like the safe option; however, performing surgery wearing a PAPR [11] may not even be possible. In addition, delaying surgery may cause a patient more harm due to disease progression. Also, as COVID-19 continues to spread, resources are getting low, and it might not be possible to provide each health care provider with proper PPE. In that case, we should start to distribute resources where they are needed most, but also where the evidence provides insight into their effectiveness.

**Table 4:** Overview of proposed questions and evidence.

Transmission route	Available evidence	Advice
Positive pressure OR^a^	Minimal	Turn off positive pressure, prepare several negative pressure ORs
Intubation/extubation	Minimal	Level III protection, should not be performed in positive pressure OR
Smoke evacuation	Minimal	Use a proper filter in a closed vacuum system
Tissue extraction	None	Use masks and screens/goggles at minimum
Desufflation of abdomen	None	Use a proper filter and a closed system

^a^OR: operating room.

The Handbook of COVID-19 Prevention and Treatment compiled by the First Affiliated Hospital, Zehjang University School of Medicine [74], has not been peer-reviewed and published in the literature; however, it does provide important lessons from previous outbreaks. The authors consider any kind of surgery to be high risk and advise level III protection during surgery (ie, surgical cap, N95 protective mask, work uniform, disposable medical protective uniform, disposable latex gloves, and a full-face PAPR device), negative pressure operating rooms and several other hygiene precautions [74].

Textbox 1 provides a summary of our recommendations.

**Summary of care advice for laparoscopic surgery during the COVID-19 pandemic. COVID-19: coronavirus disease. CT: computerized tomography. PCR: polymerase chain reaction. PPE: personal protective equipment.**

- Postpone elective surgery.
- Consider screening every patient who needs emergency surgery for COVID-19 either by PCR swab or CT scan of the thorax.
- Dedicate specific operating rooms to patients with COVID-19.
- Turn off positive pressure/create negative pressure ORs.
- Use Level III personal protective equipment during intubation and extubation.
- Consider Level III PPE but at least provide adequate mouth, face, and eye protection during surgery.
- Use proper filters and closed systems for smoke evacuation.
- Use proper filters and closed systems for CO_2_ desufflation.
- Do not perform transanal surgery.
- Consider faces as contaminated fluids.

### Comparing Open Surgery With Laparoscopic Surgery

Surgery cannot always be avoided or delayed. Should we then perform open surgery instead of laparoscopic surgery? Evidence has shown the benefits of laparoscopic surgery in many cases and for multiple indications. Should we abandon these benefits for the patient in favor of lowering the risks for health care providers? The risks related to increased OR pressure and intubation are not changed during open surgery. The smoke evacuation may be even better controlled by laparoscopy then by open surgery, and the effects of tissue extraction and desufflation are completely unknown. Cauterization may be comparable; however, dissection by sharp instruments such as scissors and use of ligatures to prevent bleeding is more common during open surgery. Blood splash risks are estimated to be 48.5% [70] in laparoscopy and 45% in open surgery [75]. Northern Italian surgeons [76] prefer laparoscopy over laparotomy, making a case for a more controlled splatter and smoke environment. In their opinion, there is no reason to perform open surgery where laparoscopy is the first choice [76].

### Preventive Measures

All studies emphasize the importance of protecting health care providers with adequate PPE whether they are performing surgery or a physical examination. However, there are diverse interpretations of how to use PPE. There are many studies examining, for example, face masks [77-79]. The debate is focused on the added value of giving the patient a mask [78] or which mask to use [79,80]. Some studies provide hospital-made protective gear solutions in case of limited resources [81] or show the added value of salt-covered masks [82]. Finally, studies that show the influence of transocular infection of influence advise the use of N95 protective gear for the eyes as well [83].

Focusing on other contamination routes, Hahn et al [84] showed that a built-in-filter trocar removes >60% of hazardous molecules during laparoscopic rectal resection, and companies are registering these trocars. SAGAS and others advise that the use of devices to filter aerosolized particles in released CO_2_ should be strongly considered and that the high pressure in the OR should be turned off or, even better, low pressure ORs should be created. A few dedicated ORs should be created for the purpose of performing emergency surgery on patients who have or are at high risk for COVID-19.

Health care providers should think logically about tissue extraction, protect themselves and OR staff, desufflate the abdomen first, and not hesitate to increase the incision slightly rather than increasing the risk of the spread of aerosols. Finally, when desufflating, use of a filter should be considered or the same system as the smoke evacuation should be used.

### Conclusions

To conclude, we would like to look forward. There is ongoing debate on the preoperative screening of asymptomatic patients and how to proceed when the peak of the crisis is over and elective surgeries can be performed again. To screen patients who are asymptomatic for COVID-19, earlier SARS-CoV-2 outbreak studies show higher sensitivity of computerized tomography (CT) scanning compared to polymerase chain reaction (PCR) swabbing [85,86]. However, more recent studies debate the actual added value in absolute numbers and the risks of false-positive outcomes even when using new classification systems [87,88]. Future studies are needed to provide proper advice about COVID-19 screening. Most of all, health care providers should use logic and common sense to protect themselves and others by performing surgery in a safe and protected environment. A global effort is being made to report on the experience and outcomes of surgical patients with COVID-19. The study protocol, registration, and details can be found at the website [89]. 

## Appendix

Multimedia Appendix 1 Search string.

Multimedia Appendix 2 Study results for anesthesia and viral infection risk.

## Abbreviations

COVID-19: coronavirus disease
CT: computerized tomography
GRADE: Grading of Recommendations, Assessment, Development, and Evaluations
HEPA: integrated high-efficiency particulate air
HPV: human papillomavirus
MERS: Middle Eastern respiratory syndrome
OR: operating room
PCR: polymerase chain reaction
PPE: personal protective equipment
SAGAS: Society of American Gastrointestinal and Endoscopic Surgeons
