# Supplementary material for: COVID-19 and Laparoscopic Surgery: Scoping Review of Current Literature and Local Expertise
Source: JMIR Public Health Surveill. 2020 Jun 23;6(2):e18928. doi: 10.2196/18928 (PMC7313384; doi:10.2196/18928)
Supplement: Multimedia Appendix 1 [file publichealth_v6i2e18928_app1.docx]

Appendix A – search strategy

Performed on 24^th^ April 2020

**Pubmed search strategy**

**General search string**

"COVID-19" [Supplementary Concept] OR "severe acute respiratory syndrome coronavirus 2" [Supplementary Concept] OR "Severe Acute Respiratory Syndrome"[Mesh] OR "SARS Virus"[Mesh] OR "COVID-19"[Supplementary Concept] OR "severe acute respiratory syndrome coronavirus 2"[Supplementary Concept] OR 2019ncov[tiab] OR 2019 ncov[tiab] OR novel coronavirus*[tiab] OR novel corona virus*[tiab] OR covid19[tiab] OR covid 19[tiab] OR sars virus[tiab] OR sars-cov[tiab] OR sars-associated coronavirus[tiab] OR severe acute respiratory syndrome coronavirus[tiab] OR severe acute respiratory syndrome virus[tiab] OR mers coronavir*[tiab] OR mers vir*[tiab] OR mers-cov[tiab] OR middle east respiratory syndrome coronavir*[tiab] OR severe acute respiratory infection*[tiab]

"Laparoscopy"[Mesh] OR "Hand-Assisted Laparoscopy"[Mesh] OR laparoscopy[tiab] OR laparoscopic surgery[tiab]


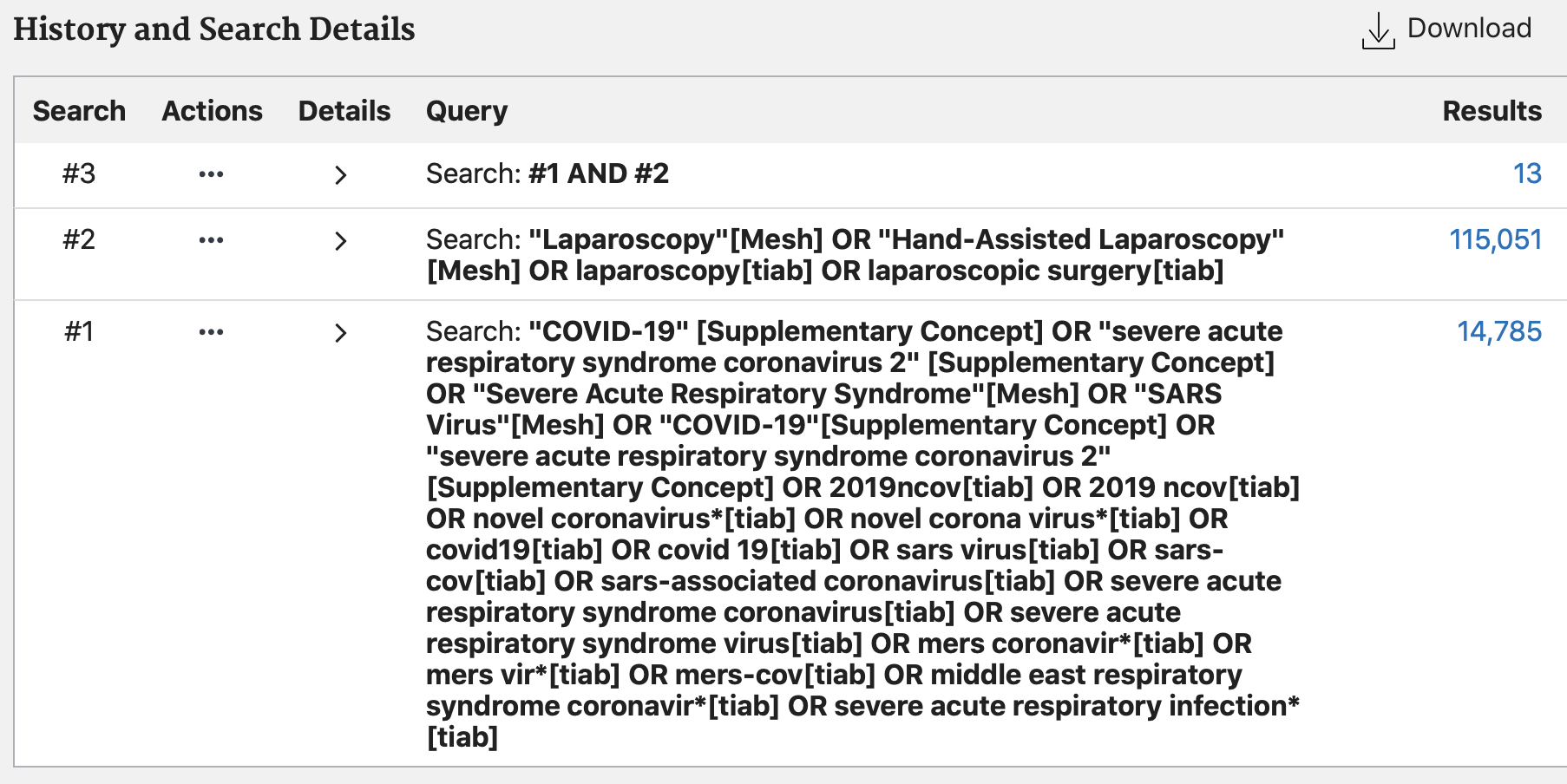


**Additional search string included:**

("Virus Diseases"[Mesh] OR "Viruses"[Mesh] OR "DNA Contamination"[Mesh] or virus[tiab] OR viral infection[tiab] OR RNA spread[tiab] OR DNA spread[tiab] OR virus contamination[tiab] OR vital contamination [tiab] OR RNA contamination[tiab] OR DNA contamination[tiab] OR virus presence[tiab] OR viral presence[tiab]

OR

"COVID-19" [Supplementary Concept] OR "severe acute respiratory syndrome coronavirus 2" [Supplementary Concept] OR "Severe Acute Respiratory Syndrome"[Mesh] OR "SARS Virus"[Mesh] OR "COVID-19"[Supplementary Concept] OR "severe acute respiratory syndrome coronavirus 2"[Supplementary Concept] OR 2019ncov[tiab] OR 2019 ncov[tiab] OR novel coronavirus*[tiab] OR novel corona virus*[tiab] OR covid19[tiab] OR covid 19[tiab] OR sars virus[tiab] OR sars-cov[tiab] OR sars-associated coronavirus[tiab] OR severe acute respiratory syndrome coronavirus[tiab] OR severe acute respiratory syndrome virus[tiab] OR mers coronavir*[tiab] OR mers vir*[tiab] OR mers-cov[tiab] OR middle east respiratory syndrome coronavir*[tiab] OR severe acute respiratory infection*[tiab])

**Search string operating room pressure**

"Operating Rooms"[Mesh] OR "Operating Room Information Systems"[Mesh] OR operation room[tiab] OR operating theater[tiab] OR surgery room[tiab] OR surgical room[tiab]

**Search string intubation and extubation**

"Intubation"[Mesh] OR "Intubation, Intratracheal"[Mesh] OR "Intubation, Gastrointestinal"[Mesh] OR "Rapid Sequence Induction and Intubation"[Mesh] OR "Anesthesia, General"[Mesh] OR detubatie[tiab] OR extubation[tiab] OR intubation[tiab] OR general anesthesia[tiab]

**Search string surgical smoke**

"Smoke"[Mesh] OR surgical smoke[tiab] OR plume*[tiab] OR tissue ablation[tiab] OR electrocautery[tiab] OR smoke plume[tiab] OR diathermy plume[tiab] OR cautery smoke[tiab] OR surgical aerosols[tiab] OR surgical bioaerosols[tiab] OR vapors[tiab] OR air contaminants[tiab]

**Search string tissue extraction**

Tissue extraction[tiab] OR tissue removal[tiab] OR tissue withdrawal[tiab]

**Search string de-sufflation after laparoscopy**

"Pneumoperitoneum"[Mesh] OR "Pneumoperitoneum, Artificial"[Mesh] OR insufflate* [tiab] OR desufflat*[tiab] OR laparoscopy gas[tiab] OR CO2 gas[tiab] OR carbonoxide gas[tiab]


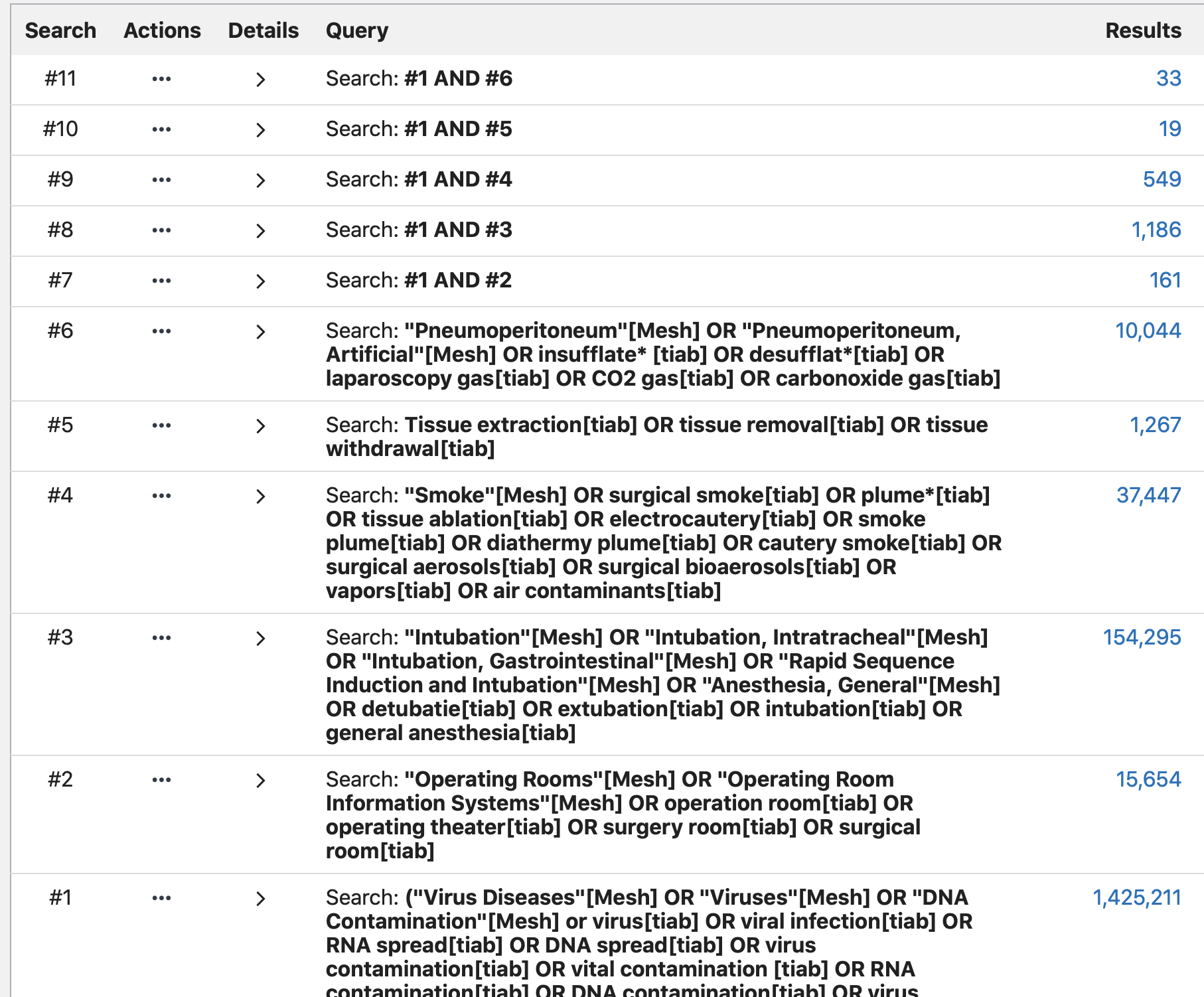


**CINAHL search string**

Same terms used as Pubmed

**Embase search string**

Same tems used as Pubmed

**Google Scholar search string**

(COVID-19 OR Corona OR severe acute respiratory syndrome coronavirus) AND (Laparoscopy OR laparoscopic surgery)

Filter since 2019 to focus on SARS-CoV-2 papers. Google scholar not used for other virusses.
