# Supplementary material for: COVID-19 and Laparoscopic Surgery: Scoping Review of Current Literature and Local Expertise
Source: JMIR Public Health Surveill. 2020 Jun 23;6(2):e18928. doi: 10.2196/18928 (PMC7313384; doi:10.2196/18928)
Supplement: Multimedia Appendix 2 [file publichealth_v6i2e18928_app2.docx]

Appendix B – study results for anesthesia and viral infection risk

*Table 1 – studies concerning SARS-CoV-2*

| **Study / Region** | **Design** | **Pathogen of evaluation** | **Period of evaluation** | **Main result / topic** |
| --- | --- | --- | --- | --- |
| Cook (1) | narrative review | SARS-CoV-2 | 2020 | purpose and use of PPE |
| U.K. |  |  |  |  |
| Wax (2) | review | SARS-CoV-2 | 2020 | anesthesia guidelines |
|  |  |  |  |  |
| Heinzerling (3) | case series | SARS-CoV-2 | 2020 | 3 of 121 HCP tested positive |
| U.S.A. |  |  |  |  |
| Meng (4) | experience paper | SARS-CoV-2 | 2020 | 29% of hospitalized COVID19 patients |
| China |  |  |  | were HCP |
| Sorbello (5) | experience paper | SARS-CoV-2 | 2020 | high level PPE. For aerosolgenerating |
| Italy |  |  |  | procedures |
| Yao (6) | experience paper | SARS-CoV-2 | 2020 | anesthesia advice for intubation |
| China |  |  |  |  |
| Zhao (7) | retrospective cohort | SARS-CoV-2 | 2020 | Anesthetic management guidelines |
| China |  |  |  |  |
| Zuo (8) | experience paper | SARS-CoV-2 | 2020 | Anesthesia guideline |
| China |  |  |  |  |
| Giwa (9) | experience paper | SARS-CoV-2 | 2020 | complete COVID19 overview |
| Italy |  |  |  |  |
| Greenland (10) | review | SARS-CoV-2 | 2020 | intubation advice |
| U.S.A. |  |  |  |  |
| Kim (11) | expert opinion | SARS-CoV-2 | 2020 | anesthesia advice |
| South Korea |  |  |  |  |
| Au Yong (12) | experience paper | SARS-CoV-2 | 2020 | intubation advice |
| Singapore |  |  |  |  |
| Zhang (13) | case series | SARS-CoV-2 | 2020 | no HCP infected |
| China |  |  |  |  |

*Table 2 – studies concerning other viruses*

| **Study / Region** | **Design** | **Pathogen of evaluation** | **Period of evaluation** | **Main result / topic** |
| --- | --- | --- | --- | --- |
| Seto (14) | review | respiratory | 2014 | only endotracheal intubation has clear |
|  |  | infections |  | evidence for aerocol-generation |
| Tran (15) | review | SARS | 2012 | intubation OR for HCP infection risk 6.6 |
|  |  |  |  |  |
| Orser (16) | experience paper | SARS | 2003 | anesthesia advice for COVID19 |
| Canada |  |  |  |  |
| Peng (17) | experience paper | SARS | 2003 | anesthesia advice vor COVID19 |
| Canada |  |  |  |  |
| Chan (18) | experience paper | SARS | 2004 | intubation is a high risk procedure |
| Hong Kong |  |  |  |  |
| Lu (19) | retrospective cohort | SARS | 2003 | 2 HCP infected due to intubation procedure |
| Taiwan |  |  |  |  |
| Tsai (20) | case study | SARS | 2003 | aerocols pass 0,3 um HEPA filters |
| Taiwan |  |  |  |  |
| Fowler (21) | retrospeective cohort | SARS | 2003 | increased contamination risk for HCP |
| Canada |  |  |  | involved in intubation |
| Christian (22) | case study | SARS | 2003 | HCP at risk during resuscitation |
| Canada |  |  |  |  |
| Caputo (23) | retrospective cohort | SARS | 2003 | 13% of HCP who intubated SARS patients |
| Canada |  |  |  | got infected |
| Cooper (24) | experience paper | SARS | 2003 | checklist for intubation |
| Canada |  |  |  |  |
| Loeb (25) | retrospective cohort | SARS | 2003 | 8 of 32 nurses infected |
| Canada |  |  |  |  |
| Pei (26) | case-control study | SARS | 2003 | intubation OR 30.8 |
| China |  |  |  |  |
| Peng (27) | experience paper | SARS | 2003 | anesthesia guidelines |
| Canada |  |  |  |  |
| Raboud (28) | retrospective cohort | SARS | 2003 | intubation OR 2.79 |
| Canada |  |  |  |  |
| Wei (29) | case series | SARS | 2003 | 3 cases of tracheostomy |
| Hong Kong |  |  |  |  |
| Ofner (30) | case series | SARS | 2003 | 17 HCP infected, mainly due to inconsisent |
| Canada |  |  |  | use of PPG and training |
| Missair (31) | review | Ebola | 2014 | anesthesia guidelines |
| West Africa |  |  |  |  |
| Trompson (32) | experiment | H1N1 | 2009 | aerocol generating procedures OR on H1N1 |
| U.K. |  |  |  | sampling not increased |

References

1. Cook TM. Personal protective equipment during the coronavirus disease (COVID) 2019 pandemic - a narrative review. Anaesthesia. 2020.

2. Wax RS, Christian MD. Practical recommendations for critical care and anesthesiology teams caring for novel coronavirus (2019-nCoV) patients. Can J Anaesth. 2020.

3. Heinzerling A, Stuckey MJ, Scheuer T, Xu K, Perkins KM, Resseger H, et al. Transmission of COVID-19 to Health Care Personnel During Exposures to a Hospitalized Patient - Solano County, California, February 2020. MMWR Morb Mortal Wkly Rep. 2020;69(15):472-6.

4. Meng L, Qiu H, Wan L, Ai Y, Xue Z, Guo Q, et al. Intubation and Ventilation amid the COVID-19 Outbreak: Wuhan's Experience. Anesthesiology. 2020.

5. Sorbello M, El-Boghdadly K, Di Giacinto I, Cataldo R, Esposito C, Falcetta S, et al. The Italian coronavirus disease 2019 outbreak: recommendations from clinical practice. Anaesthesia. 2020.

6. Yao W, Wang T, Jiang B, Gao F, Wang L, Zheng H, et al. Emergency tracheal intubation in 202 patients with COVID-19 in Wuhan, China: lessons learnt and international expert recommendations. Br J Anaesth. 2020.

7. Zhao S, Ling K, Yan H, Zhong L, Peng X, Yao S, et al. Anesthetic Management of Patients with COVID 19 Infections during Emergency Procedures. J Cardiothorac Vasc Anesth. 2020;34(5):1125-31.

8. Zuo MZ, Huang YG, Ma WH, Xue ZG, Zhang JQ, Gong YH, et al. Expert Recommendations for Tracheal Intubation in Critically ill Patients with Noval Coronavirus Disease 2019. Chin Med Sci J. 2020.

9. Giwa AL, Desai A, Duca A. Novel 2019 coronavirus SARS-CoV-2 (COVID-19): An updated overview for emergency clinicians. Emerg Med Pract. 2020;22(5):1-28.

10. Greenland JR, Michelow MD, Wang L, London MJ. COVID-19 Infection: Implications for Perioperative and Critical Care Physicians. Anesthesiology. 2020.

11. Kim HJ, Ko JS, Kim TY. Recommendations for anesthesia in patients suspected of COVID-19 Coronavirus infection. Korean J Anesthesiol. 2020;73(2):89-91.

12. Au Yong PS, Chen X. Reducing droplet spread during airway manipulation: lessons from the COVID-19 pandemic in Singapore. Br J Anaesth. 2020.

13. Zhang W, Du RH, Li B, Zheng XS, Yang XL, Hu B, et al. Molecular and serological investigation of 2019-nCoV infected patients: implication of multiple shedding routes. Emerg Microbes Infect. 2020;9(1):386-9.

14. Seto WH. Airborne transmission and precautions: facts and myths. J Hosp Infect. 2015;89(4):225-8.

15. Tran K, Cimon K, Severn M, Pessoa-Silva CL, Conly J. Aerosol generating procedures and risk of transmission of acute respiratory infections to healthcare workers: a systematic review. PLoS One. 2012;7(4):e35797.

16. Orser BA. Recommendations for Endotracheal Intubation of COVID-19 Patients. Anesth Analg. 2020;130(5):1109-10.

17. Peng PWH, Ho PL, Hota SS. Outbreak of a new coronavirus: what anaesthetists should know. Br J Anaesth. 2020;124(5):497-501.

18. Chan-Yeung M. Severe acute respiratory syndrome (SARS) and healthcare workers. Int J Occup Environ Health. 2004;10(4):421-7.

19. Lu YT, Chen PJ, Sheu CY, Liu CL. Viral load and outcome in SARS infection: the role of personal protective equipment in the emergency department. J Emerg Med. 2006;30(1):7-15.

20. Tsai YH, Wan GH, Wu YK, Tsao KC. Airborne severe acute respiratory syndrome coronavirus concentrations in a negative-pressure isolation room. Infect Control Hosp Epidemiol. 2006;27(5):523-5.

21. Fowler RA, Guest CB, Lapinsky SE, Sibbald WJ, Louie M, Tang P, et al. Transmission of severe acute respiratory syndrome during intubation and mechanical ventilation. Am J Respir Crit Care Med. 2004;169(11):1198-202.

22. Christian MD, Loutfy M, McDonald LC, Martinez KF, Ofner M, Wong T, et al. Possible SARS coronavirus transmission during cardiopulmonary resuscitation. Emerg Infect Dis. 2004;10(2):287-93.

23. Caputo KM, Byrick R, Chapman MG, Orser BJ, Orser BA. Intubation of SARS patients: infection and perspectives of healthcare workers. Can J Anaesth. 2006;53(2):122-9.

24. Cooper A, Joglekar A, Adhikari N. A practical approach to airway management in patients with SARS. CMAJ. 2003;169(8):785-7.

25. Loeb M, McGeer A, Henry B, Ofner M, Rose D, Hlywka T, et al. SARS among critical care nurses, Toronto. Emerg Infect Dis. 2004;10(2):251-5.

26. Pei LY, Gao ZC, Yang Z, Wei DG, Wang SX, Ji JM, et al. Investigation of the influencing factors on severe acute respiratory syndrome among health care workers. Beijing Da Xue Xue Bao Yi Xue Ban. 2006;38(3):271-5.

27. Peng PW, Wong DT, Bevan D, Gardam M. Infection control and anesthesia: lessons learned from the Toronto SARS outbreak. Can J Anaesth. 2003;50(10):989-97.

28. Raboud J, Shigayeva A, McGeer A, Bontovics E, Chapman M, Gravel D, et al. Risk factors for SARS transmission from patients requiring intubation: a multicentre investigation in Toronto, Canada. PLoS One. 2010;5(5):e10717.

29. Wei WI, Tuen HH, Ng RW, Lam LK. Safe tracheostomy for patients with severe acute respiratory syndrome. Laryngoscope. 2003;113(10):1777-9.

30. Ofner-Agostini M, Gravel D, McDonald LC, Lem M, Sarwal S, McGeer A, et al. Cluster of cases of severe acute respiratory syndrome among Toronto healthcare workers after implementation of infection control precautions: a case series. Infect Control Hosp Epidemiol. 2006;27(5):473-8.

31. Missair A, Marino MJ, Vu CN, Gutierrez J, Missair A, Osman B, et al. Anesthetic Implications of Ebola Patient Management: A Review of the Literature and Policies. Anesth Analg. 2015;121(3):810-21.

32. Thompson KA, Pappachan JV, Bennett AM, Mittal H, Macken S, Dove BK, et al. Influenza aerosols in UK hospitals during the H1N1 (2009) pandemic--the risk of aerosol generation during medical procedures. PLoS One. 2013;8(2):e56278.
